# The hybrid block iterative algorithm for solving the system of equilibrium problems and variational inequality problems

**DOI:** 10.1186/2193-1801-1-8

**Published:** 2012-07-13

**Authors:** Siwaporn Saewan, Poom Kumam

**Affiliations:** Department of Mathematics, Faculty of Science, King Mongkut’s University of Technology Thonburi (KMUTT), Bangmod Bangkok, 10140 Thailand

**Keywords:** Hybrid block iterative algorithm, Inverse-strongly monotone operator, Variational inequality, A system of equilibrium problem, Uniformly quasi- *ϕ*-asymptotically nonexpansive mapping, 47H05, 47H09, 47H10

## Abstract

**Abstract:**

In this paper, we construct the hybrid block iterative algorithm for finding a common element of the set of common fixed points of an infinite family of closed and uniformly quasi - *ϕ*- asymptotically nonexpansive mappings, the set of the solutions of the variational inequality for an *α*-inverse-strongly monotone operator, and the set of solutions of a system of equilibrium problems. Moreover, we obtain a strong convergence theorem for the sequence generated by this process in the framework Banach spaces. The results presented in this paper improve and generalize some well-known results in the literature.

## Introduction

In the theory of variational inequalities, variational inclusions, and equilibrium problems, the development of an efficient and implementable iterative algorithm is interesting and important. Equilibrium theory represents an important area of mathematical sciences such as optimization, operations research, game theory, financial mathematics and mechanics. Equilibrium problems include variational inequalities, optimization problems, Nash equilibria problems, saddle point problems, fixed point problems, and complementarity problems as special cases.

Let *C* be a nonempty closed convex subset of a real Banach space *E* with ∥·∥ and *E*^∗^ the dual space of *E* and *A* : *C*→*E*^∗^ be an operator. *The classical variational inequality problem* for an operator A is to find *x*^∗^ ∈ *C* such that 1.1

The set of solution of (1.1) is denoted by *VI*(*A*, *C*). Recall that let *A* : *C* → *E*^∗^ be a mapping. Then A is called

(i) *monotone* if 

(ii) *α*-*inverse-strongly monotone* if there exists a constant *α* > 0 such that 

Such a problem is connected with the convex minimization problem, the complementary problem, the problem of finding a point *x*^∗^ ∈ *E* satisfying *Ax*^∗^ = 0.

Let {*f*_*i*_}_*i*∈*Γ*_ :  be a bifunction, {*φ*_*i*_}_*i*∈*Γ*_ :  be a real-valued function, where *Γ* is an arbitrary index set. The *system of equilibrium problems*, is to find *x* ∈ *C* such that 1.2

The set of solution of (1.2) is denoted by *SEP*. If *Γ* is a singleton, then problem (1.2) reduces to the *equilibrium problem*, is to find *x* ∈ *C* such that 1.3

The set of solution of (1.3) is denoted by *EP*(*f*). The above formulation (1.3) was shown in (Blum and Oettli 1994) to cover monotone inclusion problems, saddle point problems, minimization problems, optimization problems, variational inequality problems, vector equilibrium problems, Nash equilibria in noncooperative games. In addition, there are several other problems, for example, the complementarity problem, fixed point problem and optimization problem, which can also be written in the form of an *EP*(*f*). In other words, the *EP*(*f*) is an unifying model for several problems arising in physics, engineering, science, optimization, economics, etc. In the last two decades, many papers have appeared in the literature on the existence of solutions of *EP*(*f*); see, for example (Blum and Oettli 1994; Combettes and Hirstoaga 2005) and references therein. Some solution methods have been proposed to solve the *EP*(*f*); see, for example, (Blum and Oettli 1994; Combettes and Hirstoaga 2005; Jaiboon and Kumam 2010; Katchang and Kumam 2010; Kumam 2009; Moudafi 2003; Qin et al. 2009a, 2009b, 2009c; Saewan and Kumam 2010b, 2011a, 2011b, 2011c, 2011d, 2011e, 2011f, 2011g, 2012b; Zegeye et al. 2010) and references therein.

For each *p* > 1, the *generalized duality mappingJ*_*p*_ :  is defined by 

 for all *x* ∈ *E*. In particular, *J* = *J*_2_ is called the *normalized duality mapping*. If *E* is a Hilbert space, then *J* = *I*, where *I* is the identity mapping. Consider the functional defined by 1.4

As well know that if *C* is a nonempty closed convex subset of a Hilbert space *H* and *P*_*C*_ : *H* → *C* is the metric projection of *H* onto *C*, then *P*_*C*_ is nonexpansive. This fact actually characterizes Hilbert spaces and consequently, it is not available in more general Banach spaces. It is obvious from the definition of function *ϕ* that 1.5

If *E* is a Hilbert space, then *ϕ*(*x*, *y*) = ∥*x* - *y*∥^2^, for all *x*, *y* ∈ *E*. On the author hand, the *generalized projection* (Alber 1996) *π*_*C*_ : *E* → *C* is a map that assigns to an arbitrary point *x* ∈ *E* the minimum point of the functional *ϕ*(*x*, *y*), that is, , where  is the solution to the minimization problem 1.6

existence and uniqueness of the operator *π*_*C*_ follows from the properties of the functional *ϕ*(*x*, *y* and strict monotonicity of the mapping *J* (see, for example, Alber 1996) ; Alber and Reich 1994; Cioranescu 1990; Kamimura and Takahashi 2002; Takahashi 2000).

### Remark 1.1

If *E* is a reflexive, strictly convex and smooth Banach space, then for *x*, *y* ∈ *E*, *ϕ*(*x*, *y*) = 0 if and only if *x* = *y*. It is sufficient to show that if *ϕ*(*x*, *y*) = 0 then *x* = *y*. From (1.4), we have ∥*x*∥ = ∥*y*∥. This implies that 〈*x*, *Jy*〉 = ∥*x*∥^2^ = ∥*Jy*∥^2^. From the definition of *J*, one has *Jx* = *Jy*. Therefore, we have *x* = *y*; see (Cioranescu 1990; Takahashi 2000) for more details.

Let *C* be a closed convex subset of *E*, a mapping *T* : *C* → *C* is said to be *L-Lipschitz continuous* if ∥*Tx* - *Ty* ∥ ≤ *L*∥*x* - *y*∥, ∀*x*, *y* ∈ *C* and a mapping *T* is said to be *nonexpansive* if ∥*Tx* - *Ty*∥ ≤ ∥*x* - *y*∥,∀*x*, *y* ∈ *C*. A point *x* ∈ *C* is a *fixed point*of *T* provided *Tx* = *x*. Denote by *F*(*T*) the set of fixed points of *T*; that is, *F*(*T*) = {*x* ∈ *C* : *Tx* = *x*}. Recall that a point *p* in *C* is said to be an *asymptotic fixed point* of *T*(Reich 1996) if *C* contains a sequence {*x*_*n*_} which converges weakly to *p* such that lim*n*→*∞*∥*x*_*n*_ - *Tx*_*n*_∥ = 0. The set of asymptotic fixed points of *T* will be denoted by .

A mapping *T* from *C* into itself is said to be *relatively nonexpansive* (Nilsrakoo and Saejung 2008; Su et al. 2008; Zegeye and Shahzad 2009) if  and *ϕ*(*p*, *Tx*) ≤ *ϕ*(*p*, *x*) for all *x* ∈ *C* and *p* ∈ *F*(*T*). The asymptotic behavior of a relatively nonexpansive mapping was studied in (Butnariu et al. 2001, 2003; Censor and Reich 1996). *T* is said to be *ϕ-nonexpansive*, if *ϕ*(*Tx*, *Ty*) ≤ *ϕ*(*x*, *y*) for *x*, *y* ∈ *C*. *T* is said to be *relatively quasi-nonexpansive* if *F*(*T*) ≠ *∅* and *ϕ*(*p*, *Tx*) ≤ *ϕ*(*p*, *x*) for all *x* ∈ *C* and *p* ∈ *F*(*T*). *T* is said to be *quasi-ϕ-asymptotically nonexpansive* if *F*(*T*) ≠ *∅* and there exists a real sequence {*k*_*n*_} ⊂ [1, *∞*) with *k*_*n*_ → 1 such that *ϕ*(*p*, *T*^*n*^*x*) ≤ *k*_*n*_* ϕ *(*p*, *x*) for all *n* ≥ 1 *x* ∈ *C* and *p* ∈ *F*(*T*).

We note that the class of relatively quasi-nonexpansive mappings is more general than the class of relatively nonexpansive mappings (Butnariu et al. 2001, 2003; Censor and Reich 1996; Matsushita and Takahashi 2005; Saewan et al. 2010) which requires the strong restriction: . A mapping *T* is said to be *closed* if for any sequence {*x*_*n*_} ⊂ *C* with *x*_*n*_ → *x* and *Tx*_*n*_ → *y*, then *Tx* = *y*. It is easy to know that each relatively nonexpansive mapping is closed.

### Definition 1.2

(Chang et al. 2010 (1) Let  be a sequence of mapping.  is said to be *a family of uniformly quasi-ϕ-asymptotically nonexpansive mappings*, if , and there exists a sequence {*k*_*n*_} ⊂[1, *∞*) with *k*_*n*_ → 1 such that for each *i* ≥ 1 1.7

(2) A mapping *T* : *C* → *C* is said to be *uniformly L-Lipschitz continuous*, if there exists a constant *L* > 0 such that 1.8

### Remark 1.3

It is easy to see that an *α*-inverse-strongly monotone is monotone and -Lipschitz continuous.

In 2004, Matsushita and Takahashi (2004) introduced the following iteration: a sequence {*x*_*n*_} defined by 1.9

where the initial guess element *x*_0_ ∈ *C* is arbitrary, {*α*_*n*_} is a real sequence in [0, 1], *T* is a relatively nonexpansive mapping and *π*_*C*_ denotes the generalized projection from *E* onto a closed convex subset *C* of *E*. They proved that the sequence {*x*_*n*_} converges weakly to a fixed point of *T*.

In 2005, Matsushita and Takahashi (2005) proposed the following hybrid iteration method (it is also called the CQ method) with generalized projection for relatively nonexpansive mapping T in a Banach space E: 1.10

They proved that {*x*_*n*_} converges strongly to *π*_*F*(*T*)_*x*_0_, where *π*_*F*(*T*)_ is the generalized projection from *C* onto *F*(*T*). In 2008, Iiduka and Takahashi (2008) introduced the following iterative scheme for finding a solution of the variational inequality problem for an inverse-strongly monotone operator *A* in a 2-uniformly convex and uniformly smooth Banach space *E* : *x*_1_ = *x* ∈ *C* and 1.11

for every *n* = 1, 2, 3,…, where *π*_*C*_ is the generalized metric projection from *E* onto *C*, *J* is the duality mapping from *E* into *E*^∗^ and {*λ*_*n*_} is a sequence of positive real numbers. They proved that the sequence {*x*_*n*_} generated by (1.11) converges weakly to some element of *VI*(*A*, *C*). Takahashi and Zembayashi (2008, 2009), studied the problem of finding a common element of the set of fixed points of a nonexpansive mapping and the set of solutions of an equilibrium problem in the framework of Banach spaces.

In 2009, Wattanawitoon and Kumam (2009) using the idea of Takahashi and Zembayashi (2009) extend the notion from relatively nonexpansive mappings or *ϕ*-nonexpansive mappings to two relatively quasi-nonexpansive mappings and also proved some strong convergence theorems to approximate a common fixed point of relatively quasi-nonexpansive mappings and the set of solutions of an equilibrium problen in the framework of Banach spaces. Cholamjiak (2009), proved the following iteration: 1.12

where *J* is the duality mapping on *E*. Assume that {*α*_*n*_}, {*β*_*n*_} and {*γ*_*n*_} are sequence in [0,1]. Then {*x*_*n*_} converges strongly to *q* = *π*_*F*_*x*_0_, where *F* := *F*(*T*) ∩ *F*(*S*) ∩ *EP*(*f*) ∩ *VI*(*A*, *C*).

In 2010, Saewan et al. (2010) introduced a new hybrid projection iterative scheme which is difference from the algorithm (1.12) of Cholamjiak in (2009, Theorem 3.1) for two relatively quasi-nonexpansive mappings in a Banach space. Motivated by the results of Takahashi and Zembayashi (2008); Cholamjiak and Suantai (2010) proved the strong convergence theorem by the hybrid iterative scheme for approximation of a common fixed point of countable families of relatively quasi-nonexpansive mappings in a uniformly convex and uniformly smooth Banach space: *x*_0_ ∈ *E*, , *C*_1_ = *C*1.13

Then, they proved that under certain appropriate conditions imposed on {*α*_*n*_}, and {*r*_*n*,*i*_}, the sequence {*x*_*n*_} converges strongly to .

We note that the block iterative method is a method which often used by many authors to solve the convex feasibility problem (see, Kohsaka and Takahashi 2007; Kikkawa and Takahashi 2004, etc.). In 2008, Plubtieng and Ungchittrakool (2008) established strong convergence theorems of block iterative methods for a finite family of relatively nonexpansive mappings in a Banach space by using the hybrid method in mathematical programming. Chang *et al*(2010) proposed the modified block iterative algorithm for solving the convex feasibility problems for an infinite family of closed and uniformly quasi- *ϕ*-asymptotically nonexpansive mappings, they obtained the strong convergence theorems in a Banach space. In 2010, Saewan and Kumam (2010a) obtained the result for the set of solutions of the generalized equilibrium problems and the set of common fixed points of an infinite family of closed and uniformly quasi- *ϕ*-asymptotically nonexpansive mappings in a uniformly smooth and strictly convex Banach space *E* with Kadec-Klee property.

Very recently, Qin, Cho and Kang (2009a) purposed the problem of approximating a common fixed point of two asymptotically quasi- *ϕ*-nonexpansive mappings based on hybrid projection methods. Strong convergence theorems are established in a real Banach space. Zegeye et al. (2010) introduced an iterative process which converges strongly to a common element of set of common fixed points of countably infinite family of closed relatively quasi- nonexpansive mappings, the solution set of the generalized equilibrium problem and the solution set of the variational inequality problem for an *α*-inverse strongly monotone mapping in Banach spaces.

Motivated and inspired by the work of Chang et al. (2010); Qin et al. (2009c); Takahashi and Zembayashi (2009); Wattanawitoon and Kumam (2009); Zegeye (2010); Saewan and Kumam (2010a, 2012a), we introduce a modified hybrid block projection algorithm for finding a common element of the set of the solution of the variational inequality for an *α*-inverse-strongly monotone operator, and the set of solutions of the system of equilibrium problems and the set of common fixed points of an infinite family of closed and uniformly quasi- *ϕ*-asymptotically nonexpansive mappings in a 2-uniformly convex and uniformly smooth Banach space. The results presented in this paper improve and generalize some well-known results in the literature.

## Preliminaries

A Banach space *E* is said to be *strictly convex* if  for all *x*, *y* ∈ *E* with ∥*x*∥ = ∥*y*∥ = 1 and *x* ≠ *y*. Let *U* = {*x* ∈ *E* : ∥*x*∥ = 1} be the unit sphere of *E*. Then a Banach space *E* is said to be *smooth* if the limit 

 exists for each *x*, *y* ∈ *U*. It is also said to be *uniformly smooth* if the limit is attained uniformly for *x*, *y* ∈ *U*. Let *E* be a Banach space. The *modulus of convexity* of *E* is the function *δ* :[0, 2] → [0,1] defined by 

 A Banach space *E* is *uniformly convex* if and only if *δ*(*ϵ*) > 0 for all *ϵ* ∈ (0, 2]. Let *p* be a fixed real number with *p* ≥ 2. A Banach space *E* is said to be *p-uniformly convex* if there exists a constant *c* > 0 such that *δ*(*ϵ*) ≥ *cϵ*^*p*^ for all *ϵ* ∈ [0, 2]; see (Ball et al.^1994^; Takahashi et al. 2002) for more details. Observe that every *p*-uniformly convex is uniformly convex. It is well known that a Hilbert space is *2-uniformly convex*, uniformly smooth. It is also known that if *E* is uniformly smooth, then *J* is uniformly norm-to-norm continuous on each bounded subset of *E*.

### Remark 2.1

The following basic properties can be found in Cioranescu (1990).

(i) If *E* is a uniformly smooth Banach space, then *J* is uniformly continuous on each bounded subset of *E*.
(ii) If *E* is a reflexive and strictly convex Banach space, then *J*^-1^ is norm-weak ^∗^-continuous.
(iii) If *E* is a smooth, strictly convex, and reflexive Banach space, then the normalized duality mapping  is single-valued, one-to-one, and onto.
(iv) A Banach space *E* is uniformly smooth if and only if *E*^∗^ is uniformly convex.
(v) Each uniformly convex Banach space *E* has the *Kadec-Klee property*, that is, for any sequence {*x*_*n*_} ⊂ *E*, if  and ∥*x*_*n*_∥ → ∥*x*∥, then *x*_*n*_ → *x*.

We also need the following lemmas for the proof of our main results.

### Lemma 2.2

(Beauzamy (1985); Xu (1991)). *If E be a 2-uniformly convex Banach space. Then for all x, y* ∈ *E, we have*

*where J is the normalized duality mapping of E and* 0 < *c* ≤ 1.

The best constant  in Lemma is called the *p-uniformly convex constant* of *E*.

### Lemma 2.3

(Beauzamy (1985); Zalinescu (1983)). *IfEbe a p-uniformly convex Banach space and letpbe a given real number withp* ≥ 2. *Then for allx*, *y* ∈ *E*, *j*_*x*_ ∈ *J*_*p*_(*x*) *andj*_*y*_ ∈ *J*_*p*_(*y*) 

*whereJ*_*p*_*is the generalized duality mapping ofEand**is the p-uniformly convexity constant ofE*.

### Lemma 2.4

(Kamimura and Takahashi (2002)). *Let E be a uniformly convex and smooth Banach space and let* {*x*_*n*_} *and* {*y*_*n*_} *be two sequences ofE*. *Ifϕ*(*x*_*n*_, *y*_*n*_) → 0 *and either* {*x*_*n*_} *or* {*y*_*n*_} *is bounded, then* ∥*x*_*n*_ - *y*_*n*_∥ → 0.

### Lemma 2.5

(Alber (1996)). *LetCbe a nonempty closed convex subset of a smooth Banach spaceEand x* ∈ *E*. *Then x*_0_ = *π*_*C*_*xif and only if*

### Lemma 2.6

(Alber (1996, Lemma 2.4)). *LetEbe a reflexive, strictly convex and smooth Banach space, letCbe a nonempty closed convex subset ofEand letx* ∈ *E*. *Then*

Let *E* be a reflexive, strictly convex, smooth Banach space and *J* is the duality mapping from *E* into *E*^∗^. Then *J*^-1^ is also single value, one-to-one, surjective, and it is the duality mapping from E^∗^ into *E*. We make use of the following mapping *V* studied in Alber (1996) 2.1

for all *x* ∈ *E* and *x*^∗^ ∈ *E*^∗^, that is, *V*(*x*,*x*^∗^) = *ϕ*(*x*,*J*^-1^(*x*^∗^)).

### Lemma 2.7

(Alber (1996)). *LetEbe a reflexive, strictly convex smooth Banach space and letVbe as in (*2.1). *Then*

*for allx* ∈ *Eandx*^∗^, *y*^∗^ ∈ *E*^∗^.

Let *A* be an inverse-strongly monotone mapping of *C* into *E*^∗^ which is said to be *hemicontinuous* if for all *x*, *y* ∈ *C*, the mapping *F* of [0,1] into *E*^∗^, defined by *F*(*t*) = *A*(*tx*+(1-*t*)*y*), is continuous with respect to the weak ^∗^ topology of *E*^∗^. We define by *N*_*C*_(*v*)*the normal cone* for *C* at a point *v* ∈ *C*, that is, 2.2

### Lemma 2.8

(Rockafellar (1970)). *LetCbe a nonempty, closed convex subset of a Banach spaceEandAis a monotone, hemicontinuous operator ofCintoE*^∗^. *LetB* ⊂ *E* × *E*^∗^*be an operator defined as follows:*2.3

*ThenBis maximal monotone andB*^-1^0 = *VI*(*A*, *C*).

### Lemma 2.9

(Chang et al. (2010)). *LetEbe a uniformly convex Banach space,r* > 0 *be a positive number andB*_*r*_(0) *be a closed ball ofE*. *Then, for any given sequence**and for any given sequence**of positive number with*, *there exists a continuous, strictly increasing, and convex functiong*:[0, 2*r*) → [0 ,*∞*) *withg*(0) = 0 *such that, for any positive integeri*,*jwithi* < *j*, 2.4

### Lemma 2.10

(Chang et al. (2010)). *LetEbe a real uniformly smooth and strictly convex Banach space, andCbe a nonempty closed convex subset ofE*. *LetT* : *C* → *Cbe a closed and quasi-ϕ-asymptotically nonexpansive mapping with a sequence* {*k*_*n*_} ⊂ [1, *∞*), *k*_*n*_ → 1. *ThenF*(*T*) *is a closed convex subset ofC*.

For solving the equilibrium problem for a bifunction , let us assume that *f* satisfies the following conditions:

(A1) *f*(*x*, *x*) = 0 for all *x* ∈ *C*;

(A2) *f* is monotone, i.e., *f*(*x*, *y*) + *f*(*y*, *x*) ≤ 0 for all *x*, *y* ∈ *C*;

(A3) for each *x*, *y*,*z* ∈ *C*, 

(A4) for each *x* ∈ *C*, *y* ↦ *f*(*x*, *y*) is convex and lower semi-continuous.

For example, let *A* be a continuous and monotone operator of *C* into *E*^∗^ and define 

 Then, *f* satisfies (A1)-A4). The following result is in Blum and Oettli (1994.

### Lemma 2.11

(Blum and Oettli (1994)). *LetCbe a closed convex subset of a smooth, strictly convex and reflexive Banach spaceE*, *letfbe a bifunction fromC* × *Cto**satisfying* (*A*1)- (*A*4), *and letr* > 0 *andx* ∈ *E*. *Then, there existsz* ∈ *Csuch that*

### Lemma 2.12

(Takahashi and Zembayashi (2009)). *LetCbe a closed convex subset of a uniformly smooth, strictly convex and reflexive Banach spaceEand letfbe a bifunction fromC* × *Cto**satisfying conditions* (*A*1)- (*A*4). *For allr* > 0 *andx* ∈ *E*, *define a mapping**as follows:*

*Then the following hold:*

*is single-valued;*
*is a firmly nonexpansive-type mapping (Kohsaka and Takahashi*^2008^), *that is, for allx*, *y* ∈ *E*,
4. *EP*(*f*) *is closed and convex.*

### Lemma 2.13

(Takahashi and Zembayashi (2009)). *LetCbe a closed convex subset of a smooth, strictly convex, and reflexive Banach spaceE*, *letfbe a bifunction fromC* × *Cto**satisfying* (*A*1)- (*A*4) *and letr* > 0. *Then, forx* ∈ *Eand*, 

## Strong convergence theorems

In this section, we prove the new convergence theorems for finding the set of solutions of system of equilibrium problems, the common fixed point set of a family of closed and uniformly quasi- *ϕ*-asymptotically nonexpansive mappings, and the solution set of variational inequalities for an *α*-inverse strongly monotone mapping in a *2*-uniformly convex and uniformly smooth Banach space.

### Theorem 3.1

*LetCbe a nonempty closed and convex subset of a 2-uniformly convex and uniformly smooth Banach spaceE*. *For eachj* = 1, 2, ..., *mletf*_*j*_*be a bifunction fromC* × *Cto**which satisfies conditions (A1)-(A4). LetAbe anα-inverse-strongly monotone mapping ofCintoE*^∗^*satisfying* ∥*Ay* ∥ ≤∥*Ay* - *Au*∥, ∀*y* ∈ *Candu* ∈ *VI*(*A*, *C*) ≠ *∅*. *Let**be an infinite family of closed uniformlyL*_*i*_-*Lipschitz continuous and uniformly quasi-ϕ*-*asymptotically nonexpansive mappings with a sequence* {*k*_*n*_} ⊂[1, *∞*), *k*_*n*_ → 1 *such that**is a nonempty and bounded subset inC*. *For an initial pointx*_0_ ∈ *Ewith**andC*_1_ = *C*, *define the sequence* {*x*_*n*_} *as follows:*3.1

*whereJis the duality mapping onE*, *θ*_*n*_ = sup_*q*∈*F*_(*k*_*n*_ - 1)*ϕ*(*q*, *x*_*n*_), *for eachi* ≥ 0, {*α*_*n*,*i*_} *and* {*β*_*n*_} *are sequences in* [0, 1], {*r*_*j*,*n*_} ⊂[*d*, *∞*) *for somed* > 0 *and* {*λ*_*n*_} ⊂[*a*, *b*] *for somea*,*bwith* 0 < *a* < *b* < *c*^2^*α*/2, *where**is the 2-uniformly convexity constant ofE*. *If**for alln* ≥ 0, lim inf_*n*→*∞*_*β*_*n*_(1 - *β*_*n*_) > 0 and lim inf_*n*→*∞*_*α*_*n*,0_*α*_*n*,*i*_ > 0 for all *i* ≥ 1, *then* {*x*_*n*_} *converges strongly top* ∈ *F*, *wherep* = *π*_*F*_*x*_0_.

### *Proof*.

We first show that *C*_*n*+1_ is closed and convex for each *n* ≥ 0. Clearly *C*_1_ = *C* is closed and convex. Suppose that *C*_*n*_ is closed and convex for each . Since for any *z* ∈ *C*_*n*_, we known that 

 is equivalent to 

 Hence, *C*_*n*+1_ is closed and convex.

Next, we show that *F* ⊂ *C*_*n*_ for all *n* ≥ 0. Since by the convexity of ∥ · ∥^2^, property of *ϕ*, Lemma 2.9 and by uniformly quasi- *ϕ*-asymptotically nonexpansive of *S*_*n*_ for each *q* ∈ *F* ⊂ *C*_*n*_, we have 3.2

and 3.3

It follows from Lemma 2.7, that 3.4

Since *q* ∈ *VI*(*A*, *C*) and *A* is an *α*-inverse-strongly monotone mapping, we have 3.5

By Lemma 2.2 and ∥*Ax*_*n*_∥ ≤ ∥*Ax*_*n*_ - *Aq*∥, ∀*q* ∈ *VI*(*A*, *C*), we also have 3.6

Substituting (3.5) and (3.6) into (3.4), we have 3.7

Substituting (3.7) into (3.3), we also have 3.8

and substituting (3.8) into (3.2), we obtain 3.9

Thus, this show that *q* ∈ *C*_*n*+1_ implies that *F* ⊂ *C*_*n*+1_ and hence, *F* ⊂ *C*_*n*_ for all *n* ≥ 0. This implies that the sequence {*x*_*n*_} is well defined. From definition of *C*_*n*+1_ that  and  we have 3.10

Form Lemma 2.6, it follows that 3.11

By (3.10) and (3.11), then {*ϕ*(*x*_*n*_, *x*_0_)} are nondecreasing and bounded. So, we obtain that  exists. In particular, by (1.5), the sequence {(∥*x*_*n*_∥ - ∥*x*_0_∥)^2^} is bounded. This implies {*x*_*n*_} is also bounded. We denote 3.12

Moreover, by the definition of *θ*_*n*_ and (3.12), it follows that 3.13

Next, we show that {*x*_*n*_} is a Cauchy sequence in *C*. Since , for *m* > *n*, by Lemma 2.6, we have 

 Since lim_*n*→*∞*_*ϕ*(*x*_*n*_, *x*_0_) exists and we taking *m*, *n* → *∞* then, we get *ϕ*(*x*_*m*_, *x*_*n*_) → 0. From Lemma 2.4, we have lim_*n*→*∞*_∥*x*_*m*_ - *x*_*n*_∥ = 0. Thus {*x*_*n*_} is a Cauchy sequence and by the completeness of *E* and there exist a point *p* ∈ *C* such that 3.14

Now, we claim that ∥*Ju*_*n*_ - *Jx*_*n*_∥ → 0, as *n* → *∞*. By definition of , we have 

 Since  exists, we also have 3.15

Again form Lemma 2.4, that 3.16

From *J* is uniformly norm-to-norm continuous on bounded subsets of *E*, we obtain 3.17

Since  and the definition of *C*_*n*+1_, we have 

 By (3.13) and (3.15), that 3.18

Applying Lemma 2.4, we have 3.19

Since 

 It follows from (3.23) and (3.19), that 3.20

Since *J* is uniformly norm-to-norm continuous on bounded subsets of *E*, we also have 3.21

Next, we will show that 

(*i*) We show that . It follows from definition of , we have 

 By (3.13) and (3.15), that 3.22

Form Lemma 2.4, that 3.23

Since *J* is uniformly norm-to-norm continuous, we obtain 3.24

From (3.45), we note that 

 and hence 3.25

From (3.17), (3.24) and  we get 3.26

Since *J*^-1^ is uniformly norm-to-norm continuous on bounded sets, we have 3.27

Using the triangle inequality, that 

 From (3.23) and (3.27), we have 3.28

On the other hand, we observe that 

 It follows from *θ*_*n*_ → 0, ∥*x*_*n*_ - *u*_*n*_∥ → 0 and ∥*Jx*_*n*_ - *Ju*_*n*_∥ → 0, that 3.29

From (3.2), (3.3) and (3.7), we compute 

 and hence 3.30

From (3.29), {*λ*_*n*_} ⊂[*a*, *b*] for some *a*, *b* with 0 < *a* < *b* < *c*^2^*α* / 2, liminf_*n*→*∞*_(1 - *β*_*n*_) > 0 and liminf_*n*→*∞*_*α*_*n*,0_*α*_*n*,*i*_ > 0, for *i* ≥ 0 and *k*_*n*_ → 1 as *n* → *∞*, we obtain that 3.31

From Lemma 2.6, Lemma 2.7 and (3.6), we compute 

 Applying Lemma 2.4 and (3.31) that 3.32

and we also obtain 3.33

From  is continuous, for any *i* ≥ 1 3.34

Again by the triangle inequality, we get 

 From (3.28) and (3.34), we have 3.35

By using the triangle inequality, we have 

 That is 3.36

By the assumption that ∀*i* ≥ 1, *S*_*i*_ is uniformly *L*_*i*_-Lipschitz continuous, hence we have. 3.37

By (3.23) and (3.36), it follows that . From , we have  that is . In view of closeness of *S*_*i*_, we have *S*_*i*_*p* = *p*, for all *i* ≥ 1. This imply that 

(*ii*) We show that  From Lemma 2.13 and , when , *j* = 1, 2, 3, ..., *m*, *Ωn*0 = *I*, for *q* ∈ *F*, we observe that 3.38

From (3.20), (3.21), *θ*_*n*_ → 0 as *n* → *∞* and Lemma 2.4, we get 3.39

By using triangle inequality, we have 

 From (3.20) and (3.39), we have 3.40

Again by using triangle inequality, we have 

 From (3.40),we also have 3.41

Since J is uniformly norm-to-norm continuous, we obtain 

 From *r*_*j*,*n*_ > 0 we have  as *n* → *∞*, ∀*j* = 1, 2, 3, ..., *m*, and 

 By (A2), that 

 and  we get *f*(*y*, *p*) ≤ 0 for all *y* ∈ *C*. For 0 < *t* < 1, define *y*_*t*_ = *ty* + (1 - *t*)*p*. Then *y*_*t*_ ∈ *C* which imply that *f*_*j*_(*y*_*t*_, *p*) ≤ 0. From (A1), we obtain that 

 Thus *f*_*j*_(*y*_*t*_, *y*) ≥ 0. From (A3), we have *f*_*j*_(*p*, *y*) ≥ 0 for all *y* ∈ *C* and *j* = 1, 2, 3, ..., *m*. Hence *p* ∈ *EP*(*f*_*j*_), ∀*j* = 1, 2, 3, ..., *m*. This imply that .

(*iii*) We show that *x*_*n*_ → *p* ∈ *VI*(*A*, *C*). Indeed, define *B* ⊂ *E* × *E*^∗^ by 3.42

By Lemma 2.8, *B* is maximal monotone and *B*^-1^0 = *VI*(*A*, *C*). Let (*v*, *w*) ∈ *G*(*B*). Since *w* ∈ *Bv* = *Av* + *N*_*C*_(*v*), we get *w* - *Av*∈*N*_*C*_(*v*). From *v*_*n*_ ∈ *C*, we have 3.43

On the other hand, since *v*_*n*_ = *π*_*C*_*J*^-1^(*Jx*_*n*_ - *λ*_*n*_*Ax*_*n*_). Then by Lemma 2.5, we have 

 and thus 3.44

It follows from (3.43), (3.44) and *A* is monotone and -Lipschitz continuous, that 

 where *H* = sup_*n*≥1_∥*v* - *v*_*n*_∥. Take the limit as *n* → *∞*, (3.32) and (3.33), we obtain 〈*v* - *p*, *w*〉 ≥ 0. By the maximality of *B* we have *p* ∈ *B*^-1^0, that is *p* ∈ *VI*(*A*, *C*).

Finally, we show that *p* = *π*_*F*_*x*_0_. From , we have 〈*Jx*_0_ - *Jx*_*n*_, *x*_*n*_ - *z*〉 ≥ 0, ∀*z* ∈ *C*_*n*_. Since *F* ⊂ *C*_*n*_, we also have 

 Taking limit *n* → *∞*, we obtain 

 By Lemma 2.5, we can conclude that *p* = *π*_*F*_*x*_0_ and *x*_*n*_ → *p* as *n* → *∞*. This completes the proof. □

If *S*_*i*_ = *S* for each , then Theorem 3.1 is reduced to the following Corollary.

### Corollary 3.2.

*LetCbe a nonempty closed and convex subset of a 2-uniformly convex and uniformly smooth Banach spaceE*. *For eachj* = 1, 2, ..., *mletf*_*j*_*be a bifunction fromC* × *Cto**which satisfies conditions (A1)-(A4). LetAbe anα*-*inverse-strongly monotone mapping ofCintoE*^∗^*satisfying* ∥*Ay*∥ ≤ ∥*Ay* - *Au*∥, ∀*y*∈*Candu* ∈ *VI*(*A*, *C*) ≠ *∅*. *LetS* : *C* → *Cbe a closedL*-*Lipschitz continuous and quasi-ϕ-asymptotically nonexpansive mappings with a sequence* {*k*_*n*_} ⊂ [1,*∞*), *k*_*n*_ → 1 *such that**is a nonempty and bounded subset in C. For an initial pointx*_0_ ∈ *Ewith**andC*_1_=*C*, *we define the sequence* {*x*_*n*_} *as follows:*3.45

*whereJis the duality mapping onE*, *θ*_*n*_ = sup_*q*∈*F*_(*k*_*n*_ - 1)*ϕ*(*q*, *x*_*n*_), {*α*_*n*_}, {*β*_*n*_} *are sequences in [0, 1]*, {*r*_*j*,*n*_} ⊂[*d*, *∞*) *for somed* > 0 *and* {*λ*_*n*_} ⊂[*a*, *b*] *for somea*, *bwith* 0 < *a* < *b* < *c*^2^*α* / 2, where *is the 2-uniformly convexity constant ofE*. *If* lim inf_*n*→*∞*_(1 - *β*_*n*_) > 0 *and* lim inf_*n*→*∞*_*α*_*n*_(1 - *α*_*n*_) > 0, *then* {*x*_*n*_} *converges strongly top* ∈ *F*, *wherep* = *π*_*F*_*x*_0_.

For a special case that *i* = 1, 2, we can obtain the following results on a pair of quasi- *ϕ*-asymptotically nonexpansive mappings immediately from Theorem 3.1.

### Corollary 3.3.

Let *C* be a nonempty closed and convex subset of a 2-uniformly convex and uniformly smooth Banach space *E*. For each *j* = 1, 2, ..., *m* let *f*_*j*_ be a bifunction from *C* × *C* to  which satisfies conditions (A1)-(A4). Let *A* be an *α*-inverse-strongly monotone mapping of *C* into *E*^∗^ satisfying ∥*Ay*∥ ≤ ∥*Ay* - *Au*∥, ∀*y* ∈ *C* and *u* ∈ *VI*(*A*, *C*) ≠ *∅*. Let *S*, *T* : *C* → *C* be two closed quasi- *ϕ*-asymptotically nonexpansive mappings and *L*_*S*_, *L*_*T*_-Lipschitz continuous, respectively with a sequence {*k*_*n*_} ⊂ [1,*∞*), *k*_*n*_ → 1 such that  is a nonempty and bounded subset in *C*. For an initial point *x*_0_∈*E* with  and *C*_1_ = *C*, we define the sequence {*x*_*n*_} as follows: 3.46

where *J* is the duality mapping on *E*, *θ*_*n*_ = sup_*q*∈*F*_(*k*_*n*_ - 1)*ϕ*(*q*, *x*_*n*_), {*α*_*n*_}, { *β*_*n*_}, {*γ*_*n*_} and {*δ*_*n*_} are sequences in [0, 1], {*r*_*j*,*n*_} ⊂[*d*, *∞*) for some *d* > 0 and {*λ*_*n*_} ⊂[*a*, *b*] for some *a*, *b* with 0 < *a* < *b* < *c*^2^*α* / 2, where  is the 2-uniformly convexity constant of *E*. If *α*_*n*_ + *β*_*n*_ + *γ*_*n*_ = 1 for all *n* ≥ 0 and lim inf_*n*→*∞*_*α*_*n*_*β*_*n*_ > 0, lim inf_*n*→*∞*_*α*_*n*_*γ*_*n*_ > 0, lim inf_*n*→*∞*_*β*_*n*_*γ*_*n*_ > 0 and lim inf_*n*→*∞*_*δ*_*n*_(1 - *δ*_*n*_) > 0, then {*x*_*n*_} converges strongly to *p* ∈ *F*, where *p* = *π*_*F*_*x*_0_.

### Corollary 3.4.

Let *C* be a nonempty closed and convex subset of a 2-uniformly convex and uniformly smooth Banach space *E*. For each *j* = 1, 2, ..., *m* let *f*_*j*_ be a bifunction from *C* × *C* to  which satisfies conditions (A1)-(A4). Let *A* be an *α*-inverse-strongly monotone mapping of *C* into *E*^∗^ satisfying ∥*Ay*∥ ≤ ∥*Ay* - *Au*∥, ∀*y* ∈ *C* and *u* ∈ *VI*(*A*, *C*) ≠ *∅*. Let  be an infinite family of closed quasi- *ϕ*- nonexpansive mappings such that  For an initial point *x*_0_∈*E* with  and *C*_1_ = *C*, we define the sequence {*x*_*n*_} as follows: 3.47

where *J* is the duality mapping on *E*, {*α*_*n*,*i*_} and {*β*_*n*_} are sequences in [0, 1], {*r*_*j*,*n*_} ⊂[*d*, *∞*) for some *d* > 0 and {*λ*_*n*_} ⊂[*a*, *b*] for some *a*, *b* with 0 < *a* < *b* < *c*^2^*α* / 2, where  is the 2-uniformly convexity constant of *E*. If  for all *n* ≥ 0, lim inf_*n*→*∞*_(1 - *β*_*n*_) > 0 and lim inf_*n*→*∞*_*α*_*n*,0_*α*_*n*,*i*_ > 0 for all *i* ≥ 1, then {*x*_*n*_} converges strongly to *p* ∈ *F*, where *p* = *π*_*F*_*x*_0_.

### Proof

Since  is an infinite family of closed quasi- *ϕ*-nonexpansive mappings, it is an infinite family of closed and uniformly quasi- *ϕ*-asymptotically nonexpansive mappings with sequence *k*_*n*_ = 1. Hence the conditions appearing in Theorem 3.1*F* is a bounded subset in *C* and for each *i* ≥ 1,*S*_*i*_ is uniformly *L*_*i*_-Lipschitz continuous are of no use here. By virtue of the closeness of mapping *S*_*i*_ for each *i* ≥ 1, it yields that *p* ∈ *F*(*S*_*i*_) for each *i* ≥ 1, that is, . Therefore all conditions in Theorem 3.1 are satisfied. The conclusion of Corollary 3.4 is obtained from Theorem 3.1 immediately. □

### Corollary 3.5

(Zegeye 2010, Theorem 3.2) *Let Cbe a nonempty closed and convex subset of a 2-uniformly convex and uniformly smooth Banach spaceE*. *Letfbe a bifunction fromC* × *Cto**satisfying* (*A*1)- (*A*4). *LetA* be an *α-inverse-strongly monotone mapping ofCintoE*^∗^*satisfying* ∥*Ay*∥ ≤ ∥*Ay* - *Au*∥, ∀*y* ∈ *Candu* ∈ *VI*(*A*, *C*) ≠ *∅*. *Let**be a finite family of closed quasi-ϕ-nonexpansive mappings such that**For an initial pointx*_0_ ∈ *Ewith**andC*_1_ = *C*, *we define the sequence* {*x*_*n*_} *as follows:*3.48

*whereJis the duality mapping onE*, {*α*_*n*,*i*_} *is sequence in [0, 1],* {*r*_*n*_} ⊂[*d*, *∞*) *for somed* > 0 *and* {*λ*_*n*_} ⊂[*a*, *b*] *for somea*, *bwith* 0 < *a* < *b* < *c*^2^*α* / 2, *where**is the 2-uniformly convexity constant ofE*. *Ifα*_*i*_ ∈ (0, 1) *such that*, *then* {*x*_*n*_} *converges strongly top* ∈ *F*, *wherep* = *π*_*F*_*x*_0_.

### Corollary 3.6

*LetCbe a nonempty closed and convex subset of a uniformly convex and uniformly smooth Banach spaceE*. *Letfbe a bifunction fromC* × *Cto**satisfying* (*A*1)- (*A*4). *Let**be an infinite family of closed and uniformly quasi-ϕ-asymptotically nonexpansive mappings with a sequence* {*k*_*n*_} ⊂[1, *∞*), *k*_*n*_ → 1 *and uniformlyL*_*i*_*-Lipschitz continuous such that**is a nonempty and bounded subset inC*. *For an initial pointx*_0_ ∈ *Ewith**andC*_1_ = *C*, *we define the sequence* {*x*_*n*_} *as follows:*3.49

*whereJis the duality mapping onE*, *θ*_*n*_ = sup_*q*∈*F*_(*k*_*n*_ - 1)*ϕ*(*q*, *x*_*n*_), {*α*_*n*,*i*_} *is sequence in [0, 1]*, {*r*_*n*_} ⊂[*a*, *∞*) *for somea* > 0. *If**for alln* ≥ 0 *and* lim inf_*n*→*∞*_*α*_*n*,0_*α*_*n*,*i*_ > 0 *for alli* ≥ 1, *then* {*x*_*n*_} *converges strongly top* ∈ *F*, *wherep* = *π*_*F*_*x*_0_.

## Deduced to Hilbert spaces

If *E* = *H*, a Hilbert space, then *E* is 2-uniformly convex (we can choose *c* = 1) and uniformly smooth real Banach space and closed relatively quasi-nonexpansive map reduces to closed quasi-nonexpansive map. Moreover, *J* = *I*, identity operator on *H* and *π*_*C*_ = *P*_*C*_, projection mapping from *H* into *C*. Thus, the following corollaries hold.

### Theorem 4.1

*LetCbe a nonempty closed and convex subset of a Hilbert spaceH*. *For eachj* = 1, 2, ..., *mletf*_*j*_*be a bifunction fromC* × *Cto**which satisfies conditions (A1)-(A4). LetAbe anα-inverse-strongly monotone mapping ofCintoHsatisfying* ∥*Ay*∥ ≤ ∥*Ay* - *Au* ∥ , ∀*y* ∈ *Candu* ∈ *VI*(*A*, *C*) ≠ *∅*. *Let**be an infinite family of closed and uniformly quasi- ϕ-asymptotically nonexpansive mappings with a sequence* {*k*_*n*_} ⊂[1, *∞*), *k*_*n*_ → 1 and uniformly *L*_*i*_-*Lipschitz continuous such that**is a nonempty and bounded subset inC*. *For an initial pointx*_0_ ∈ *Hwith**andC*_1_ = *C*, *define the sequence* {*x*_*n*_} *as follows:*4.1

*whereθ*_*n*_ = sup_*q*∈*F*_(*k*_*n*_ - 1)∥*q* - *x*_*n*_∥, {*α*_*n*,*i*_} *is sequence in [0, 1],* {*r*_*j*,*n*_} ⊂[*a*, *∞*) *for somea* > 0 *and* {*λ*_*n*_} ⊂[*a*, *b*] *for somea*, *bwith* 0 < *a* < *b* < *α*/2. *If**for alln* ≥ 0 *and* lim inf_*n*→*∞*_*α*_*n*,0_*α*_*n*,*i*_ > 0 *for alli* ≥ 1, *then* {*x*_*n*_} *converges strongly top* ∈ *F*, *wherep* = *π*_*F*_*x*_0_.

### Remark 4.2

Theorem 4.1 improve and extend the Corollary 3.7 in Zegeye (2010) in the aspect for the mappings, we extend the mappings from a finite family of closed relatively quasi-nonexpansive mappings to more general an infinite family of closed and uniformly quasi- *ϕ*-asymptotically nonexpansive mappings.

## Zero points of an inverse-strongly monotone operator

Next, we consider the problem of finding a zero point of an inverse-strongly monotone operator of *E* into *E*^∗^. Assume that *A* satisfies the conditions:

(C1)*A* is *α*-inverse-strongly monotone,

(C2) *A*^-1^0 = {*u* ∈ *E* : *Au* = 0} ≠ *∅*.

### Theorem 5.1

*LetCbe a nonempty closed and convex subset of a 2-uniformly convex and uniformly smooth Banach spaceE*. *For eachj* = 1, 2, ..., *mletf*_*j*_*be a bifunction fromC* × *Cto**which satisfies conditions (A1)-(A4). LetAbe an operator ofEintoE*^∗^*satisfying (C1) and (C2). Let**be an infinite family of closed uniformlyL*_*i*_-*Lipschitz continuous and uniformly quasi-ϕ-asymptotically nonexpansive mappings with a sequence* {*k*_*n*_} ⊂[1,*∞*), *k*_*n*_ → 1 *such that*

*is a nonempty and bounded subset inC*. *For an initial pointx*_0_ ∈ *Ewith**andC*_1_ = *C*, *define the sequence* {*x*_*n*_} *as follows:*5.1

*whereJis the duality mapping onE*, *θ*_*n*_ = sup_*q*∈*F*_(*k*_*n*_ - 1)*ϕ*(*q*,*x*_*n*_), *for eachi* ≥0, {*α*_*n*,*i*_} *and* {*β*_*n*_} *are sequences in [0, 1]*, {*r*_*j*,*n*_}⊂[*d*, *∞*) for some *d* > 0 *and* {*λ*_*n*_} ⊂[*a*, *b*] *for somea*, *bwith* 0 < *a* < *b* < *c*^2^*α* / 2, *where**is the 2-uniformly convexity constant ofE*. *If**for alln* ≥ 0, lim inf_*n* → *∞*_(1 - *β*_*n*_) > 0 and lim inf_*n*→*∞*_*α*_*n*,0_*α*_*n*,*i*_ > 0 *for alli* ≥ 1, *then* {*x*_*n*_} *converges strongly top*∈*F*, *wherep* = *π*_*F*_*x*_0_.

### *Proof*.

Setting *C* = *E* in Corollary 3.4, we also get *π*_*E*_ = *I*. We also have *VI*(*A*, *C*) = *VI*(*A*, *E*) = {*x* ∈ *E* : *Ax* = 0} ≠ *∅* and then the condition ∥*Ay*∥ ≤ ∥*Ay* - *Au*∥ holds for all *y* ∈ *E* and *u* ∈ *A*^-1^0. So, we obtain the result. □

## Complementarity problems

Let *K* be a nonempty, closed convex cone in *E*. We define the *polarK*^∗^ of *K* as follows: 6.1

If *A* : *K* → *E*^∗^ is an operator, then an element *u* ∈ *K* is called a solution of the *complementarity problem* (Takahashi 2000) if 6.2

The set of solutions of the complementarity problem is denoted by *CP*(*A*, *K*).

### Theorem 6.1

*LetKbe a nonempty closed and convex subset of a 2-uniformly convex and uniformly smooth Banach spaceE*. *For eachj* = 1, 2, ..., *mletf*_*j*_*be a bifunction fromC* × *Cto**which satisfies conditions (A1)-(A4). LetAbe anα-inverse-strongly monotone mapping ofKintoE*^∗^*satisfying* ∥*Ay*∥ ≤ ∥*Ay* - *Au*∥, ∀*y* ∈ *Kandu* ∈ *CP*(*A*, *K*) ≠ *∅*. *Let**be an infinite family of closed uniformlyL*_*i*_*-Lipschitz continuous and uniformly quasi-ϕ-asymptotically nonexpansive mappings with a sequence* {*k*_*n*_} ⊂[1,*∞*), *k*_*n*_ → 1 *such that**is a nonempty and bounded subset inK*. *For an initial pointx*_0_ ∈ *Ewith**andK*_1_ = *K*, *we define the sequence* {*x*_*n*_} *as follows:*6.3

*whereJis the duality mapping onE*, *θ*_*n*_ = sup_*q*∈*F*_(*k*_*n*_ - 1)*ϕ*(*q*,*x*_*n*_), *for eachi* ≥ 0, {*α*_*n*,*i*_} *and* {*β*_*n*_} *are sequences in [0, 1],* {*r*_*j*,*n*_} ⊂[*d*, *∞*) *for somed* > 0 *and* {*λ*_*n*_} ⊂[*a*, *b*] *for somea*,*bwith* 0 < *a* < *b* < *c*^2^*α* / 2, *where**is the 2-uniformly convexity constant ofE*. *If**for alln* ≥ 0, lim inf_*n* → *∞*_(1 - *β*_*n*_) > 0 and lim inf_*n*→*∞*_*α*_*n*,0_*α*_*n*,*i*_ > 0 for all *i* ≥ 1, *then* {*x*_*n*_} *converges strongly top* ∈ *F*, *wherep* = *π*_*F*_*x*_0_.

### *Proof*.

As in the proof of Takahashi in (Takahashi 2000, Lemma 7.11), we get that *VI*(*A*, *K*) = *CP*(*A*, *K*). So, we obtain the result. □
